# Association between Self-Perception of Chewing, Chewing Behavior, and the Presence of Gastrointestinal Symptoms in Candidates for Bariatric Surgery

**DOI:** 10.3390/nu16081096

**Published:** 2024-04-09

**Authors:** Flávia Luciana Pinheiro de Souza Pinto Martins, Millena Borges Inete, Yasmym Dannielle do Espírito Santo Souza, Rafaela Lorena Viana Costa, Rafaelle Dias Gabbay, Tainá Martins Moraes, Vanessa Vieira Lourenço Costa, Carla Cristina Paiva Paracampo, Luiz Carlos de Albuquerque, Daniela Lopes Gomes

**Affiliations:** 1 Graduate Program in Neuroscience and Behavior, Behavior Theory and Research Center, Federal University of Pará, Belém 66075-110, Brazil; flavia-luciana@hotmail.com (F.L.P.d.S.P.M.); millenainete@gmail.com (M.B.I.); dannielleyasmym@gmail.com (Y.D.d.E.S.S.); rafaelaviananutri@gmail.com (R.L.V.C.); rafaellediasgabbay@gmail.com (R.D.G.); tainamartmo@gmail.com (T.M.M.); cparacampo@gmail.com (C.C.P.P.); lcalbu58@gmail.com (L.C.d.A.); 2 Faculty of Nutrition, Federal University of Pará, Belém 66075-110, Brazil; vlourencocosta@hotmail.com

**Keywords:** bariatric surgery, obesity, chewing, gastrointestinal symptoms, speech therapy

## Abstract

Given the changes in the digestive tract post-bariatric surgery, adapting to a new pattern of eating behavior becomes crucial, with special attention to the specifics of chewing mechanics. This study aimed to investigate the association between self-perception of chewing, chewing behavior, and the presence of gastrointestinal symptoms in preoperative patients undergoing bariatric surgery. Sixty adult candidates for bariatric surgery at a public hospital in Belém (Brazil) were analyzed. Participants predominantly exhibited unilateral chewing patterns (91.6%), a fast chewing rhythm (73.3%), a large food bolus (80%), liquid intake during meals (36.7%), and 41.7% reported that chewing could cause some issue. Significant associations were found between the perception of causing problems and chewing scarcity (*p* = 0.006), diarrhea (*p* = 0.004), absence of slow chewing (*p* = 0.048), and frequent cutting of food with front teeth (*p* = 0.034). These findings reveal a relationship between the perception of chewing problems and chewing scarcity, presence of diarrhea, and fast chewing.

## 1. Introduction

Obesity is a chronic global disease with multifactorial etiology, showing an increasing incidence and becoming one of the greatest public health challenges worldwide over the last three decades [1]. In Brazil, statistics indicate that 57.2% of the population is overweight [2].

According to the WHO [3], obesity is characterized by the accumulation of body fat, causing health impairments, and is classified based on the Body Mass Index (BMI) above 30 kg/m^2^ with associated mortality risk. Its treatment involves approaches such as nutritional therapy, use of weight-loss medications, and physical exercise. However, many patients do not respond satisfactorily to conventional clinical treatment, necessitating surgical intervention, known as bariatric surgery [4]. Regardless of the type of treatment indicated, an interdisciplinary follow-up during the process is essential [5].

Bariatric surgery comprises techniques aiming to induce body mass reduction, enhance quality of life, and improve obesity-related comorbidities [6]. In Brazil, this treatment is recommended for individuals with a BMI above 40 kg/m^2^, irrespective of comorbidities, or for individuals with a BMI greater than 35 kg/m^2^ associated with comorbidities [7].

Surgical treatment does not negate the need to adopt a healthy lifestyle, which should be maintained long-term post-surgery, as weight loss depends on changes in behavior and eating habits [8,9].

Due to changes in the digestive tract after bariatric surgery, adapting to a new eating behavior pattern becomes crucial, paying attention to the specifics of chewing mechanics [10]. Chewing corresponds to the initial phase of the digestive process, constituting a functional system comprising anatomical and physiological structures that allow for optimal chewing movements [11].

Chewing involves a variable sequence of chewing cycles, initiated by sensory receptors activated by food [12]. Through this sensory input, the chewing-generating centers—specifically, the trigeminal sensory–motor complex and the trigeminal motor nucleus—controlling the chewing muscles, adjust parameters such as the number of cycles, muscle force, and jaw movements based on the transformation of food consistency [13].

The primary function of chewing is to break down food into smaller particles, mixing them with saliva. Saliva moistens the ingested food, while salivary enzymes bind to it, forming a cohesive, slippery bolus safe for swallowing and digestion [14].

For chewing to be considered correct or appropriate, specific characteristics during the chewing process are necessary. Initially, incision should occur using the incisors to cut the food. Subsequently, the food should be moved to the premolar teeth for grinding. Finally, the food should reach the molars to be pulverized, assisted by saliva, forming the food bolus [15]. Additionally, chewing should happen bilaterally and alternately, involving vertical and rotational jaw movements, with a slow rhythm, and with closed lips to assist the tongue during grinding [16].

Well-chewed food is considered to aid in the digestion process since digestive enzymes act on the surface of food particles. When a person chews thoroughly, food is broken down into smaller particles, increasing the surface area exposed to digestive enzymes [17,18].

On the other hand, inadequate chewing can lead to food reaching the stomach disorganized and with large volumes of air, causing undesirable gastrointestinal symptoms such as flatulence, heartburn, reflux, nausea, and vomiting [19,20,21].

There are suggestions indicating that when a person chews, sensory information from the orofacial region is transmitted to the trigeminal sensory–motor complex and the trigeminal motor nucleus by trigeminal afferent neurons, chemically adjusting motor actions to absorb nutrients until satiety is achieved [22]. Proper chewing patterns are necessary for individuals with obesity due to its impact on digestion and possibly on hunger and satiety perception, potentially associated with gastrointestinal symptoms and weight gain [23,24].

Studies have suggested a relationship between chewing and weight gain [25,26], characterized by rapid chewing in individuals with severe obesity and/or those who have undergone bariatric surgery [27,28,29]. Some studies have shown that chewing quickly and less frequently is associated with obesity [30,31]. Others have indicated that better chewing behavior and reduced bite size increase satiety and anticipate meal termination [32,33,34], slowing down chewing speed and reducing portion size, thereby contributing to weight loss [35,36,37].

In this context, our hypothesis is that individuals with severe obesity exhibit inadequate chewing behavior, which may not be self-perceived and is associated with the presence of gastrointestinal symptoms. Therefore, the aim of this study was to investigate the association between chewing behavior, self-perception of chewing, and the presence of gastrointestinal symptoms in candidates for bariatric surgery.

## 2. Materials and Methods

### 2.1. Participants

The research had a cross-sectional, descriptive, and analytical design. Sixty adults between 20 and 60 years old, candidates for bariatric surgery, residing in the metropolitan area of Belém (Brazil) and in rural areas of the state, were selected by convenience. They were being followed-up at the Endocrinology outpatient clinic at Jean Bitar Hospital and agreed to participate in the research, signing the Informed Consent Form (ICF). Individuals under 18 or over 60 years old, patients with facial or occlusal deformities, hearing impairments, cognitive impairment, prior neurological conditions (such as stroke, traumatic brain injury, among others), who used any medication that could cause gastrointestinal symptoms, and patients who had received speech therapy treatment or guidance on chewing were excluded from the study.

### 2.2. Instruments

#### 2.2.1. Data Collection Form

Developed by researchers, this form was used for the sociodemographic and clinical characterization of participants. It included name, gender, age, education level, occupation, place of residence, marital status, family income, Body Mass Index (BMI), and clinical status (associated chronic diseases and problems). Participants had to answer yes or no to the presence of symptoms after eating such as vomiting, dyspepsia, reflux, flatulence, heartburn, gastralgia, diarrhea, and constipation. Additionally, it inquired whether participants had received guidance on how to avoid these symptoms, and if so, the nature of this guidance.

#### 2.2.2. Chewing Evaluation Protocol

This instrument encompassed topics related to functional chewing assessment conducted by a speech therapist, such as incision or cutting of food by the front teeth, lateral teeth, or absence of incision; unilateral chewing pattern—right, left, or bilateral; systematic or absent lip closure; slow (more than 40 s) or fast (less than 30 s) chewing rhythm; an average of 15 to 20 masticatory cycles would be considered appropriate for the quantity of the food chosen; rotational or vertical jaw movements; food bolus size—small (portion of food) or large (whole food); and presence or absence of excessive chewing, chewing scarcity, and pain while chewing, need for liquids during chewing, and noise in the temporomandibular joint (TMJ). Participants were asked to eat a piece of French bread, approximately one-sixth of the bread, while a speech therapist observed their chewing. The document was adapted from validated protocols by Gonçalves and Chehter [38] and the orofacial myofunctional evaluation protocol MBGR, named after its authors’ surnames—Marchesan, Berrentin-Felix, Genaro, and Rehder [39].

#### 2.2.3. Self-Perception Chewing Form

A questionnaire designed by a speech therapist researcher consisting of 13 specific questions regarding chewing behavior. For instance, whether the participant puts small pieces of food in their mouth, chews slowly, chews on both sides, cuts food with front teeth, closes their mouth while chewing, experiences pain while chewing, tends to drink liquids to swallow food, experiences heartburn after eating, experiences dyspepsia and gastric fullness after eating, has episodes of vomiting, experiences choking episodes during meals, takes a long time to finish a meal, whether the participant believes their chewing is causing them any problems, and if they had any difficulty eating meat. Participants read and completed each question about their chewing behaviors together with the researcher, selecting one of four response options (always, frequently, sometimes, never). The symptom grades were summed up in the data analysis.

### 2.3. Procedure

The 60 pre-bariatric surgery participants were individually assessed by the responsible researcher. Initially, they completed the data collection form, providing sociodemographic, clinical, and information regarding their knowledge about chewing behavior, aiming to characterize the sample. The researcher previously assessed the dentition of all participants to ensure that the absence of teeth or the use of dental prostheses did not confuse the interpretation of the results. Afterward, participants underwent an evaluation of chewing behavior using the chewing assessment protocol. Subsequently, they completed the self-perception chewing questionnaire.

The assessment using the chewing protocol was solely conducted by the speech therapist researcher in a private setting, with participants seated during the evaluation. The researcher handed each participant a piece of French bread, approximately ten grams of the loaf and provided a glass of water in case the participant needed liquids during the test. The researcher said something like, “Please, you may start eating”. Three (3) offers of food (French bread) were made to obtain an average of chewing cycles and observe rhythm, scarcity, or excess of chewing. French bread was chosen as it allows for easy visualization of chewing and does not cause atypical reactions, ensuring good comparability, acceptance, and visibility of chewing [38].

The participant’s chewing was recorded based on observed behavior, following the items in the chewing assessment protocol. Subsequently, the researcher provided the self-perception chewing questionnaire with specific questions about chewing behavior. These questions were read together with the researcher and answered by the participant.

### 2.4. Data Analysis

Statistical analysis was performed using SPSS software, version 24.0. Results were expressed in absolute frequency and proportion. The Chi-square test of independence with adjusted residual analysis was applied, considering a statistical significance level of *p* > 0.05 for the comparison of all study variables.

### 2.5. Ethical Considerations

The execution of this study was conducted in accordance with the Declaration of Helsinki of 1975, revised in 2013, and approved by the research ethics committee involving human subjects at the Tropical Medicine Center at the Federal University of Pará, under protocol 4.052.573-CEP/NMT. The research complies with the ethical–legal precepts (autonomy, non-maleficence, beneficence, and justice) recommended in Resolution No. 466/2012 regarding research involving human subjects by the Brazilian National Health Council, and its execution was approved by the teaching and research sector of the Jean Bitar Regional Hospital.

## 3. Results

### 3.1. Sociodemographic Profile

In this study, 60 pre-bariatric surgery patients were evaluated, aged between 20 and 60 years old, with a median age of 39.6 (±10.4) years. The majority were female (n = 58; 96.7%), married (n = 38; 63.3%), had completed high school education (n = 26; 43.3%), had a family income of one to three minimum wages (n = 34; 56.7%), resided in the metropolitan area of Belém (n = 41; 68.3%), and their average BMI was 46.2 ± 9 kg/m^2^. Of the participants, 15% had previously received guidance on gastrointestinal symptom prevention strategies at some point in their lives (Table 1). 

### 3.2. Self-Perception of Chewing

In relation to participants’ responses to the self-perception chewing questionnaire, 43.3% reported never putting small pieces of food in their mouths, 56.7% stated never chewing slowly, and 50% mentioned never chewing on both sides. Furthermore, 53.3% reported always making incisions in food with their front teeth, 70% always closed their mouths during chewing, and 58.3% never felt pain during chewing. Concerning meal duration, 71.7% reported never taking a long time to finish meals. Regarding the perception that chewing caused them any issues, 58.3% responded never, while 35% said it sometimes caused problems, and approximately 83% drank liquids during meals (Table 2).

### 3.3. Chewing Behavior

During the chewing assessment, the main behaviors exhibited by the participants included always making incisions in the food bolus with their front teeth (53.3%). This aligns with what was reported in the self-perception chewing questionnaire, where 53.3% reported always cutting food with their front teeth.

Another notable characteristic was the left-sided unilateral chewing pattern (53.3%), followed by the right-sided unilateral pattern (38.3%), consistent with the participants’ reports in the self-perception chewing questionnaire, where 50% claimed to never chew on both sides.

Additionally, systematic lip closure was observed in 83.3% of participants. In the questionnaire, 70% of participants reported always closing their mouths during chewing (Table 2 and Table 3).

The results obtained in the assessment of chewing rhythm showed that 73.3% had a fast rhythm, similar to the self-perception findings where 71.3% reported not taking much time for meals. As for the observed mandibular movements, rotational movements were observed in 58.3% of participants (Table 2 and Table 3).

Regarding the size of the food bolus placed in the mouth during the assessment, it was noted that 80% put large pieces of food in their mouths (Table 3). This differs from the reported information by the participants, as only 43.3% mentioned always putting large pieces of food in their mouths (Table 2).

Regarding liquid intake during meals, 36.7% drank liquids during the meal in the assessment. Additionally, noise during chewing was present in 31.7%, snoring in 60%, and sleep apnea in 26.7% of the evaluated participants (Table 3).

When comparing self-perception of chewing with the assessed chewing behavior, only 41.7% of participants reported that chewing could cause any problems. However, 73.3% presented incorrect chewing patterns during the assessment, 80% put large pieces of food in their mouths, 83.3% had a unilateral chewing pattern, and 43.3% performed few chews. Only the ability to maintain lip closure during meals was similar in self-perception and assessment.

### 3.4. Gastrointestinal Symptoms

Regarding the presence of gastrointestinal symptoms, 58.3% of participants reported the presence of flatulence, 40% reported gastroesophageal reflux, 30% reported feeling gastric fullness frequently, 28.3% reported feeling postprandial heartburn frequently, 21.7% reported diarrhea, and 18.3% reported gastralgia (Table 4).

### 3.5. Relationship between Self-Perception of Chewing, Chewing Characteristics, and the Presence of Gastrointestinal Symptoms

Regarding the relationship between self-perception of chewing, the observed chewing characteristics by the speech therapist during evaluations, and the presence of gastrointestinal symptoms, it was found that perceiving chewing as problematic was associated with a low frequency of chewing movements (*p* = 0.006). Having diarrhea was associated with perceiving chewing as problematic (*p* = 0.004). Never chewing slowly was associated with the perception that chewing causes problems, while chewing slowly was associated with the perception that chewing does not cause problems (*p* = 0.048). Cutting food with the front teeth always was associated with the perception that chewing does not cause problems, whereas frequently cutting food with the front teeth was associated with the perception that chewing causes problems (*p* = 0.034). Moreover, experiencing frequent choking during meals was associated with the perception that chewing causes problems (*p* = 0.035) (Table 5).

## 4. Discussion

The present study investigated the association between chewing behavior, self-perception of chewing, and the presence of gastrointestinal symptoms in candidates for bariatric surgery. 

A higher frequency of consuming large food particles was observed both in the participants’ reports and in the conducted evaluations. These findings align with those from a study by Isabel et al. [40], which identified that individuals with obesity ingested larger food particles both during chewing and swallowing tests, indicating reduced chewing performance and a greater tendency to swallow larger particles.

The ingestion of large food particles may lead to inefficient digestion when compared to thorough chewing, potentially causing prolonged intestinal transit, and resulting in gastrointestinal discomforts such as irritation, abdominal distension, pain, nausea, and vomiting [41]. In more severe cases, it may lead to intestinal obstruction [42]. Additionally, it can have a negative impact on body weight and eating behavior [43].

Most participants reported chewing quickly, which aligns with the proportion of rapid chewing identified in the assessment. Another study involving participants with obesity also found that they chewed less and consumed food more rapidly compared to non-overweight participants [44], with a prevalence of rapid chewing among participants with obesity similar to the proportion found in this study.

These results differ from those found by White et al. [45], who did not identify significant differences in chewing speed between individuals with and without excess weight, suggesting that individuals with normal weight may also exhibit inappropriate chewing behavior and that weight alone is not the sole determinant of this practice. Therefore, it becomes necessary to study other factors, such as psychological issues and lifestyle, which may be associated with inadequate chewing patterns.

Individual sensitivity, gastrointestinal tract motility, intestinal microbiota, immune response, and the state and functioning of the intestinal barrier can be influenced by food intake and habits such as smoking and alcohol consumption. These factors are often associated with the appearance of gastrointestinal symptoms. The most investigated food groups in this context are lipids, fermentable carbohydrates, caffeine, milk and derivatives, dietary fibers, probiotics, and gluten [17].

The rapid chewing behavior in obese patients and candidates for bariatric surgery is well documented in the literature [46,47,48], but studies comparing the assessment of this behavior with self-perception of chewing in these individuals have not been identified, highlighting the contribution of this research to the literature.

Encouraging slow chewing may contribute to reducing self-reported hunger sensations, reducing the amount of food consumed, and preventing excessive flatulence [49]. This practice optimizes the digestion process, allowing the stomach to adequately break down food particles for the subsequent segments, increasing satiety and insulin sensitivity, which positively impacts glucose absorption [50,51]. It also proves beneficial for pre- and post-surgical weight loss and for establishing healthy eating behavior [23,52].

The behavior of always consuming liquids during meals was reported by 25% of the participants, similar to the findings of Moraes et al. [53], who found that about 29.63% of individuals with obesity also exhibited this behavior. However, evidence suggests that this practice should be discouraged as it is generally associated with symptoms of poor digestion, vomiting, and dumping syndrome [54,55,56].

It was found that most participants (91.6%) exhibited a unilateral chewing pattern, contrasting with self-reported perceptions where only 50% reported having this pattern. In a study involving individuals with obesity, 66% reported adopting a unilateral chewing pattern [53]. Pedroni-Pereira et al. also found a higher prevalence of unilateral chewing in overweight women, which aligns with the results of our study. The overweight group also showed more alterations in myofunctional and orofacial aspects when compared to eutrophic participants [57].

The unilateral chewing pattern results in greater development of the jaw on the side opposite to chewing and greater development of the maxilla on the side where chewing occurs [58]. The ideal and physiologically adequate chewing pattern is bilateral alternation, where chewing cycles alternate between the right and left sides [59]. This pattern allows for the proper distribution of chewing force, alternating work and rest, promoting muscle and functional synchrony and balance, stimulating the development and/or maintenance of dental arches, and occlusal stability [12].

Furthermore, the incision of food should occur using the incisors or front teeth, as they play the role of cutting food, functioning as levers to break it down [12,60,61]. This practice was identified in both the reports and evaluations of our participants.

A high frequency of gastrointestinal symptoms was observed among the participants in this study, with a higher frequency of gastroesophageal reflux, flatulence, postprandial heartburn, and sensation of gastric fullness. This can be partially explained by the nutritional profile of this population, as obesity is considered a risk factor for gastrointestinal disorders due to associated anatomical and physiological alterations [62,63,64,65,66]. Based solely on the collected data, it is not possible to clearly identify what may have generated such behaviors. However, some suggestions found in the literature can be provided for the conduct of future research. 

The habit of eating quickly may be associated with the sensation of gastric fullness and excessive flatulence [67,68,69]. The onset of these symptoms may also be linked to dietary patterns, with high consumption of ultra-processed foods and a scarcity of dietary fiber [70,71].

An association was also observed between reported perception of chewing problems and digestive symptoms such as diarrhea and choking. Choking incidents may be related to haste during eating [72], inadequate chewing, and the ingestion of large food particles, which hinders swallowing [73,74].

A possible explanation for the association between chewing problems and the occurrence of diarrhea is the rapid arrival of undigested food in the small intestine, the release of gastrointestinal hormones, and the water imbalance in the intestinal compartment caused by hyperosmolar molecules [54,55,56].

This analysis suggests the necessity of providing preoperative guidance emphasizing changes in eating behavior, such as instructions related to chewing and swallowing [75]. These instructions play an important role in preventing undesirable gastrointestinal symptoms and contribute to the success of the surgery [76].

While some studies have suggested that changes in chewing capacity may indirectly influence the onset of gastrointestinal disorders, there is a scarcity of literature on the relationship between chewing assessment and gastrointestinal symptoms [76,77,78,79].

It is assumed that the perception that chewing can cause problems influences individuals’ food choices, leading them to prefer foods that are easier to chew, even if they are not necessarily the healthiest. This preference can negatively impact gastrointestinal health, contributing to the manifestation of gastrointestinal symptoms.

Regarding the discrepancy between self-perception of chewing and evaluated chewing behavior, a possible hypothesis would be the difficulty in self-assessing chewing behavior. Normally, in certain verbal settings (such as restaurants and other circumstances where eating can be evaluated), people are exposed to justifications for chewing with their mouths closed. In more specific verbal environments (such as speech therapy, nutrition, theater, etiquette courses, among others), a person may be exposed to justifications for them to perceive their own chewing and to be aware of whether it is correct. However, in general, people are not encouraged to closely evaluate their chewing behavior in their daily lives unless confronted with specific questions or a professional assessment.

Therefore, in everyday life, activities like chewing, swallowing, and other oral functions are often automatic and, therefore, rarely subjected to conscious evaluation. Individuals, in general, do not usually reflect on the details of the chewing process unless prompted to do so, such as in a clinical or research context, where observation and reporting of these activities become necessary. Additionally, lack of education about the importance of proper chewing may contribute to a distorted self-perception. This highlights the importance of greater awareness regarding the significance of chewing for overall health.

In this regard, encouraging proper chewing behavior in candidates for bariatric surgery could be facilitated through promises related to weight loss, health, and other well-being components, according to the Theory of Justifications and Immediate Consequences Control (TJC Theory). This theory allows us to understand how environmental variables, including treatment rules, justifications, and immediate consequences, influence and maintain behavior [80,81,82].

According to the TJC Theory, promises of weight loss, body aesthetics, health, well-being, and happiness function as justifications for the specified behavior outlined by treatment rules to occur and be maintained [80,81,82]. In this context, treatment rules may include guidance on how to chew correctly, with the justification that it is essential for proper digestion and to avoid complications.

Future research could explore how these promises can be more effectively integrated into preoperative strategies to optimize the outcomes of bariatric surgery. This study contributes to the literature on chewing behavior by comparatively analyzing self-perception and chewing mechanics in candidates for bariatric surgery, a gap not identified in the existing literature on the subject. 

Despite the relevance of these questions, the present study has some limitations, such as its cross-sectional design, which only allows testing associations and describing certain characteristics based on the specific sample. Moreover, due to the context of the COVID-19 pandemic during the research, recruiting participants was challenging, thereby highlighting the need for future studies with a larger and more representative sample. 

Another limitation of the study was the challenge in accurately assessing the chewing pattern, particularly during systematic lip closure. Therefore, it is necessary and relevant to conduct studies that employ imaging examinations to accurately evaluate chewing movements.

It was also not possible to recruit a control group due to pandemic restrictions at the time of data collection; however, the comparison of chewing mechanics between people with and without obesity is already documented in the literature. Despite the difficulties, the obtained results are valuable and contribute to clarifying the relationship between chewing, self-perception of chewing, and gastrointestinal symptoms, providing an initial foundation for the development of future studies, especially those of longitudinal and experimental nature.

## 5. Conclusions

Participants exhibited a predominantly unilateral chewing pattern, a rapid chewing pace, rotary mandibular movements, an insertion of a bulky food bolus into the mouth, and the need for liquid intake during meals. Additionally, an association was found between the perception of chewing problems and a scarcity of chewing, occurrences of diarrhea, rapid chewing, and choking incidents. 

Considering these findings, it underscores the importance of patients receiving guidance during pre-surgical consultations regarding necessary changes in eating behavior, emphasizing the justifications for following instructions related to chewing and swallowing. Attention to these aspects is crucial to avoid undesirable gastrointestinal symptoms, aiding in weight loss, and contributing to the success of treatment in patients undergoing bariatric surgery.

## Figures and Tables

**Table 1:** Sociodemographic profile of candidates for bariatric surgery.

	n (%)	*p*-Value *
**Gender**		
Female	58 (96.7%)	<0.001
Male	2 (3.3%)	
**Marital Status**		
Single	20 (33.3%)	<0.001
Married	38 (63.3%)	
Divorced	1 (1.7%)	
Widowed	1 (1.7%)	
**Education Levels**		
Complete Elementary Education	1 (1.7%)	<0.001
Incomplete Elementary Education	3 (5%)	
Complete High School Education	26 (43.3%)	
Incomplete High School Education	5 (8.3%)	
Complete Higher Education	13 (21.7%)	
Incomplete Higher Education	12 (20%)	
Family Income **		
≤USD 253.73	12 (20%)	<0.001
>USD 253.74 to USD 763.63	34 (56.7%)	
>USD 763.63 to USD 1.268.65	12 (20%)	
>USD 1.268.65	2 (3.33%)	
**City of Residence**		
Belém and Metropolitan Region I	41 (68.3%)	<0.001
Interior of the State of Pará	19 (31.7%)	
**Receipt of guidance to prevent gastrointestinal symptoms**		
Yes	9 (15%)	<0.001
No	51 (85%)	
Age in years (mean ± SD ***)	39.6 ± 10.4	
**BMI in kg/m^2^ (mean ± SD)**	46.2 ± 9	

* Chi-square test. ** Values regarding the Brazilian minimum wage converted to US dollar amounts in the year 2020. *** Standard Deviation.

**Table 2:** Characterization of self-perception regarding chewing among candidates for bariatric surgery.

	n (%)	*p*-Value *
**Putting small pieces of food in the mouth**		
Always	17 (28.3%)	<0.001
Frequently	1 (1.7%)	
Sometimes	16 (26.7%)	
Never	26 (43.3%)	
**Chewing slowly**		
Always	6 (10%)	<0.001
Frequently	3 (5%)	
Sometimes	17 (28.3%)	
Never	34 (56.7%)	
**Chewing on both sides**		
Always	11 (18.3%)	<0.001
Frequently	9 (15%)	
Sometimes	10 (16.7%)	
Never	30 (50%)	
**Cutting food with front teeth**		
Always	32 (53.3%)	<0.001
Frequently	3 (5%)	
Sometimes	10 (16.7%)	
Never	15 (25%)	
**Closing the mouth while chewing**		
Always	42 (70%)	<0.001
Frequently	2 (3.3%)	
Sometimes	11 (18.3%)	
Never	5 (8.3%)	
**Feeling pain while chewing**		
Always	1 (1,7%)	<0.001
Frequently	3 (5%)	
Sometimes	21 (35%)	
Never	35 (58.3%)	
**Drinking liquids during the meal**		
Always	15 (25%)	0.002
Frequently	8 (13.3%)	
Sometimes	27 (45%)	
Never	10 (16.7%)	
**Taking a long time to finish meals**		
Always	2 (3.3%)	<0.001
Frequently	1 (1.7%)	
Sometimes	14 (23.3%)	
Never	43 (71.7%)	
**Considering chewing causes any issue**		
Always	1 (1.7%)	<0.001
Frequently	3 (5%)	
Sometimes	21 (35%)	
Never	35 (58.3%)	

* Chi-square test.

**Table 3:** Evaluation of chewing behavior among candidates for bariatric surgery.

	n (%)	*p*-Value *
**Incision or cutting of food**		
Front Teeth	32 (53.3%)	0.002
Side Teeth	18 (30%)	
Absence or use of cutlery	10 (16.7%)	
**Chewing pattern**		
Bilateral	5 (8.3%)	<0.001
Right Unilateral	23 (38.3%)	
Left Unilateral	32 (53.3%)	
**Lip closure**		
Systematic	53 (88.3%)	<0.001
Non-systematic	7 (11.7%)	
**Chewing rhythm**		
Slow	16 (26.7%)	<0.001
Fast	44 (73.3%)	
**Jaw movement**		
Rotational	35 (58.3%)	0.197
Vertical	25 (41.7%)	
**Food bolus size**		
Small	12 (20%)	<0.001
Big	48 (80%)	
**Excessive chewing**		
Yes	32 (53.3%)	0.606
No	28 (46.7%)	
**Scarcity of chewing**		
Yes	26 (43.3%)	0.302
No	34 (56.7%)	
**Need for liquid after the meal**		
Yes	22 (36.7%)	0.039
No	34 (56.7%)	
**Presence of pain while chewing**		
Absent	26 (42.6%)	<0.001
Present	5 (8.2%)	
**Presence of noise while chewing**		
Absent	41 (68.3%)	0.005
Present	19 (31.7%)	
**Snoring**		
Yes	32 (53.3%)	0.121
No	24 (40%)	
**Sleep apnea**		
Yes	16 (26.7%)	<0.001
No	44 (73.3%)	

* Chi-square Test.

**Table 4:** Characterization of the frequency of gastrointestinal symptoms among candidates for bariatric surgery.

	n (%)	*p*-Value *
**Emesis**		
Yes	3 (5%)	<0.001
No	57 (95%)	
**Gastroesophageal reflux**		
Yes	24 (40%)	0.121
No	36 (60%)	
**Flatulence**		
Yes	35 (58.3%)	0.197
No	25 (41.7%)	
**Diarrhea**		
Yes	13 (21.7%)	<0.001
No	47 (78.3%)	
**Constipation**		
Yes	5 (8.3%)	<0.001
No	55 (91.7%)	
**Gastralgia**		
Yes	11 (18.3%)	<0.001
No	49 (81.7%)	
**Postprandial heartburn**		
Always	0 (0%)	0.522
Frequently	17 (28.3%)	
Sometimes	24 (40%)	
Never	19 (31.7%)	
**Postprandial gastric fullness**		
Always	1 (1.7%)	<0.001
Frequently	18 (30%)	
Sometimes	24 (40%)	
Never	17 (28.3%)	
**Choking during meals**		
Always	0 (0%)	<0.001
Frequently	3 (5%)	
Sometimes	16 (26.7%)	
Never	41 (68.3%)	

* Chi-square test.

**Table 5:** Statistically significant relationships between self-perception of chewing, chewing characteristics, and the presence of gastrointestinal symptoms in participants undergoing bariatric surgery candidacy.

	n (%)		*p*-Value *
	Self-perception of chewing		
	Cause problems	Does not cause problems	
**Scarcity of chewing**			
Yes	16 (26.7%) (+)	10 (16.7%) (−)	0.006
No	9 (15%) (−)	25 (41.7%) (+)	
**Presence of diarrhea**			
Yes	10 (16.7%) (+)	3 (5%) (−)	0.004
No	15 (25%) (−)	32 (53.3%) (+)	
**Slow chewing**			
Always	2 (3.3%)	4 (6.7%)	0.048
Frequently	1 (1.7%)	2 (3.3%)	
Sometimes	3 (5%) (−)	14 (23.3%) (+)	
Never	19 (31.7%) (+)	15 (25%) (−)	
**Cuts the food with the front teeth**			
Always	9 (15%) (−)	23 (38.3%) (+)	0.034
Frequently	3 (5%) (+)	0 (0%) (−)	
Sometimes	4 (6.7%)	6 (10%)	
Never	9 (15%)	6 (10%)	
**Choking during meals**			
Always	0 (0%)	0 (0%)	0.035
Frequently	3 (5%) (+)	0 (0%) (−)	
Sometimes	8 (13.3%)	8 (13.3%)	
Never	14 (23.3%)	27 (45%)	

* Pearson’s chi-squared test with adjusted residual analysis, where (+) indicates a positive association in the category and (−) indicates a negative association in the analysis category. This table includes only statistically significant associations, considering *p* < 0.05.

## Data Availability

Data are available upon request due to restrictions aimed at preserving the privacy of the participants. The data presented in this study are available upon request to the project’s research coordinator. The data are not publicly available due to ethical considerations regarding the preservation of participants’ identities.
